# Development and validation of a nomogram for predicting persistent inflammation, immunosuppression, and catabolism syndrome in trauma patients

**DOI:** 10.3389/fmed.2023.1249724

**Published:** 2023-08-24

**Authors:** Ligang Xu, Zhaofeng Kang, Dongfang Wang, Yukun Liu, Chuntao Wang, Zhanfei Li, Xiangjun Bai, Yuchang Wang

**Affiliations:** ^1^Division of Trauma Surgery, Emergency Surgery and Surgical Critical, Tongji Trauma Center, Wuhan, China; ^2^Department of Emergency and Critical Care Medicine, Tongji Hospital, Tongji Medical College, Huazhong University of Science and Technology, Wuhan, China; ^3^Department of Plastic and Cosmetic Surgery, Tongji Hospital, Tongji Medical College, Huazhong University of Science and Technology, Wuhan, China

**Keywords:** nomogram, persistent inflammation, immunosuppression, catabolism syndrome, trauma score, trauma prediction, ICUAW

## Abstract

**Background:**

Persistent Inflammation, Immunosuppression, and Catabolism Syndrome (PIICS) is a significant contributor to adverse long-term outcomes in severe trauma patients.

**Objective:**

The objective of this study was to establish and validate a PIICS predictive model in severe trauma patients, providing a practical tool for early clinical prediction.

**Patients and methods:**

Adult severe trauma patients with an Injury Severity Score (ISS) of ≥16, admitted between October 2020 and December 2022, were randomly divided into a training set and a validation set in a 7:3 ratio. Patients were classified into PIICS and non-PIICS groups based on diagnostic criteria. LASSO regression was used to select appropriate variables for constructing the prognostic model. A logistic regression model was developed and presented in the form of a nomogram. The performance of the model was evaluated using calibration and ROC curves.

**Results:**

A total of 215 patients were included, consisting of 155 males (72.1%) and 60 females (27.9%), with a median age of 51 years (range: 38–59). NRS2002, ISS, APACHE II, and SOFA scores were selected using LASSO regression to construct the prognostic model. The AUC of the ROC analysis for the predictive model in the validation set was 0.84 (95% CI 0.72–0.95). The Hosmer-Lemeshow test in the validation set yielded a χ^2^ value of 14.74, with a value of *p* of 0.098.

**Conclusion:**

An accurate and easily implementable PIICS risk prediction model was established. It can enhance risk stratification during hospitalization for severe trauma patients, providing a novel approach for prognostic prediction.

## Introduction

The majority of severe trauma patients require treatment in the intensive care unit (ICU). Due to improvements in clinical treatment and care in recent years, the mortality rate of these patients has decreased (1, 2). However, surviving patients often experience prolonged stays in the ICU and enter a state of Chronic Critical Illness (CCI) (1, 3). In 2012, Gentile et al. coined the term Post-Intensive Care Syndrome (PIICS) and defined its clinical determinants as Persistent Inflammation, Immunosuppression, and Catabolism Syndrome. These determinants include prolonged hospitalization (>14 days), inflammation (C-reactive protein levels >150 μg/dL), immune suppression (lymphocyte count <800/μL), and catabolism (weight loss >10% during hospitalization or BMI <18.5 kg/m^2^) (1, 4). PIICS is characterized by prolonged dysregulation of the inflammatory response, immune dysfunction, and catabolic state, resulting in a range of adverse outcomes, including infection, organ dysfunction, and impaired wound healing (2, 5). Early identification and prediction of PIICS in trauma patients are crucial for optimizing patient management and improving long-term prognosis.

The development and validation of predictive models specifically designed for trauma patients can assist healthcare professionals in identifying high-risk individuals and preventing PIICS-related complications (6). Predictive models can integrate clinical and demographic variables, provide early risk stratification, and facilitate targeted interventions. Currently, there is a primary focus on predictive models for PIICS in critically ill patients in different clinical settings, such as sepsis and major surgeries (6, 7). However, trauma patients present unique challenges and characteristics (5). The pathological mechanisms underlying the development of PIICS in trauma patients are not yet clear, and factors such as the severity of the injury, anatomical location, and surgical interventions may significantly influence the risk and trajectory of PIICS (5, 8–10). Therefore, there is a need to establish a robust dataset encompassing diverse demographic characteristics, injury severity, clinical variables, and biomarker measurements, and employ advanced statistical techniques and machine learning algorithms to derive predictive models with good discriminative and calibration abilities, enhancing their applicability in clinical practice.

This study aims to develop and validate a predictive model specifically for severe trauma patients to predict PIICS. The model will incorporate clinical and injury-related variables to provide physicians with a reliable tool for assessing the risk of PIICS in individual trauma patients. By identifying high-risk patients early on, healthcare professionals can implement targeted interventions to modulate dysregulated inflammatory responses and alleviate the occurrence and progression of PIICS-related complications. This effective prediction approach can be utilized to reduce the risk of PIICS-related complications and improve patient recovery and long-term health.

## Materials and methods

A prospective survey was conducted from October 2020 to September 2022 to collect data from severe trauma patients aged 18 and above admitted to the Trauma Intensive Care Unit (ICU) of Tongji Hospital, Huazhong University of Science and Technology School of Medicine. Clinical data within 24 h of admission were assessed and recorded, including age, gender, mechanism of injury (MOI), body mass index (BMI), Injury Severity Score (ISS), Nutritional Risk Screening 2002 (NRS 2002), Sequential Organ Failure Assessment (SOFA) score, and Acute Physiology and Chronic Health Evaluation II score (APACHE II score). Laboratory examinations at admission included hemoglobin (g/L), lymphocyte count (*10^9^/L), albumin (g/L), and lactate (mmol/L). The BMI was calculated as weight divided by height squared (kg/m^2^). 14 days after admission, the relevant indicators of PIICS were recorded and evaluated. Specific indicators include inflammation (C-reactive protein level), immune suppression (lymphocyte count), and catabolism (weight loss during hospitalization or BMI). This research plan has been approved by the Medical Ethics Committee of Tongji Hospital, Tongji Medical College, Huazhong University of Science and Technology (approval number: TJ-IRB20230214). According to the guidelines, the study satisfied the conditions to waive the requirement for informed consent from individual participants. Therefore, informed consent was waived by Medical Ethics Committee of Tongji Hospital, Tongji Medical College, Huazhong University of Science and Technology. All procedures were carried out following relevant guidelines and regulations.

### Definition of PIICS

According to Gentile et al.’s study in 2012, PIICS was defined as Persistent Inflammation, Immunosuppression, and Catabolism Syndrome (1). The clinical determinants of PIICS were prolonged hospitalization (>14 days), inflammation (C-reactive protein levels >150 μg/dL), immune suppression (lymphocyte count <800/μL), and catabolism (weight loss >10% during hospitalization or BMI <18.5 kg/m^2^) (1, 2).

### Statistical analysis

Patients were divided into PIICS and non-PIICS groups based on the occurrence of PIICS during hospitalization. Normally distributed data were presented as means and standard deviations (SDs), while non-normally distributed data were presented as medians and interquartile ranges (IQRs). Differences between groups were evaluated using unpaired *t*-tests or the Mann–Whitney *U* test for continuous variables. Frequency tables were generated for categorical variables and analyzed using chi-square or Fisher’s exact tests. Univariate logistic regression was performed to explore risk factors for adverse outcomes during hospitalization. Factors with a value of *p* <0.2 were entered into the multivariate regression model. The results of the final model were expressed as hazard ratios (HRs) with 95% confidence intervals (CIs).

Patients were randomly allocated to a training set and a validation set in a 7:3 ratio. In the training set, the least absolute shrinkage and selection operator (LASSO) regression with 10-fold cross-validation was used to select the appropriate variables, with λ set at one standard error (SE). A logistic regression model was developed to predict the occurrence of adverse outcomes during hospitalization, and the predictive performance of the prognostic model was internally validated in the validation set. The final model was presented graphically. Goodness of fit was assessed using the Hosmer-Lemeshow test. Calibration curves and receiver operating characteristic (ROC) curves were plotted to analyze the discriminative ability and calibration of the model. Statistical analysis was performed using R version 4.0.2 with relevant packages.

## Results

### Descriptive data

A total of 215 patients were included, with 155 males (72.1%) and 60 females (27.9%). The median age was 51 years (38–59). The most common mechanisms of injury were traffic accidents in 136 cases (63.3%), followed by falls in 34 cases (15.8%). The median BMI was 24.22 kg/m^2^ (10.90–30.11). The top three body regions with the most severe injuries were the head and neck (26.5%), abdomen (20.1%), and chest (20%). The median NRS 2002 and ISS scores were 3 (1–3) and 25 (19–33), respectively.

### Factors associated with PIICS

Based on the occurrence of PIICS, patients were divided into the PIICS group (79 cases) and the non-PIICS group (136 cases). There were statistically significant differences between the PIICS and non-PIICS groups in terms of NRS2002 score, ISS score, APACHE II score, and SOFA score (*p* < 0.05) (Tables 1, 2).

**Table 1:** Comparison of characteristics between PIICS and No-PIICS patients.

Variables	PIICS cases (*n* = 79)	No-PIICS cases (*n* = 136)	Total patients (*n* = 215)	Value of *p*
Gender				0.181
Male	52	103	155	
Female	27	33	60	
Age (year)	53 (43–60)	49 (37–58)	51 (38–59)	0.122
MOI (*n*)				0.296
Vehicle collision	46	90	136	
Fall	12	22	34	
Others	21	24	45	
Injury region				0.573
Head	20	37	57	
Thorax	16	28	44	
Abdomen	17	28	45	
Pelvis	10	8	18	
Spine	6	10	16	
Extremity	10	25	35	
Pelvis	10	8	18	
BMI	23.75 (20.50–33.05)	24.06 (21.73–29.35)	24.22 (10.90–30.11)	0.426
NRS2002 score	3 (2–4)	2 (1–3)	3 (1–3)	0.0002
ISS score	27 (21–34)	22 (17–31)	25 (19–33)	0.003
APACHE II score	11 (9–15)	11 (7–11)	11 (8–13)	0.002
SOFA score	5 (2–7)	3 (2–4)	3 (2–5)	0.0008
Lymphocyte (*10^9/L)	0.92 (0.61–1.355)	0.91 (0.65–1.31)	0.91 (0.63–1.34)	0.813
Hb (g/L)	96 (82–105)	103 (89–116)	98 (85–114)	0.022
Alb (g/L)	31.8 ± 5.5	33.4 ± 4.7	32.4 ± 5.1	0.062
Serum creatinine (umol/L)	57 (46–83)	64 (53–76)	63 (50–76)	0.256
hs-CRP (mg/L)	55.2 (28.5–109.5)	48 (12.4–77.8)	33.8 (29.5–37.1)	0.084
Emergency surgery	17	25	42	0.719

**Table 2:** Baseline characteristics of 215 participants.

Variables	Training set (*n* = 150)	Validation set (*n* = 65)	Total patients (*n* = 215)	Value of *p*
Gender				0.7601
Male	109	46	155	
Female	41	19	60	
Age (year)	51 (38–60)	50 (39–57)	51 (38–59)	0.5041
BMI	24.33 (20.98–28.38)	23.90 (21.20–31.43)	24.22 (10.90–30.11)	0.7307
NRS2002 score	3 (1–3)	3 (2–4)	3 (1–3)	0.0458
ISS score	24 (19–29)	26 (20–34)	25 (19–33)	0.4140
APACHE II score	11 (8–13)	11 (8–13)	11 (8–13)	0.9681
SOFA score	3 (2–5)	3 (2–7)	3 (2–5)	0.7315
Lymphocyte (*10^9/L)	0.91 (0.64–1.35)	0.95 (0.62–1.31)	0.91 (0.63–1.34)	0.9318
Hb (g/L)	98 (85–112)	99 (88–117)	98 (85–114)	0.6785
Alb (g/L)	32.9 ± 4.7	32.7 ± 5.9	32.8 ± 5.1	0.0555
Serum creatinine (umol/L)	63 (51–78)	61 (48–73)	63 (50–76)	0.2561
hs-CRP (mg/L)	35.0 (29.6–38.2)	33.8 (29.8–35.8)	33.8 (29.5–37.1)	0.0430
Emergency surgery	32	10	42	0.4077

### Logistic regression analysis

Univariate logistic regression analysis revealed that independent factors associated with the occurrence of PIICS included NRS2002 score (HR 1.46, 95% CI 1.19–1.82), ISS score (HR 1.05, 95% CI 1.02–1.08), APACHE II score (HR 1.11, 95% CI 1.04–1.20), SOFA score (HR 1.17, 95% CI 1.07–1.32), albumin (HR 0.94, 95% CI 0.88–0.99), and hs-CRP (HR 1.01, 95% CI 1.00–1.01). Multivariate logistic regression analysis identified NRS2002 score (HR 1.29, 95% CI 1.02–1.65) as an independent factor associated with PIICS (Table 3).

**Table 3:** Logistic regression for factors associated with PIICS.

Variable	Univariate analysis			Multivariate analysis		
HR	95%CI	*p* value	HR	95%CI	*p* value
Gender	0.63	0.32–1.25	0.1823			
Age	1.01	0.99–1.04	0.2657			
BMI	0.99	0.96–1.04	0.8516			
NRS2002	1.46	1.19–1.82	0.0004	1.29	1.02–1.65	0.0356
ISS	1.05	1.02–1.08	0.0033	1.02	0.98–1.06	0.2014
APACHE II score	1.11	1.04–1.20	0.0042	1.08	0.99–1.17	0.0645
SOFA score	1.17	1.07–1.32	0.0021	0.97	0.90–1.04	0.2329
Lymphocyte	0.90	0.53–1.49	0.6881			
Hb	0.99	0.98–1.00	0.2148			
Alb	0.94	0.88–0.99	0.0494	0.97	0.90–1.04	0.3812
Serum creatinine	1.00	0.99–0.99	0.9894			
hs-CRP	1.01	1.00–1.01	0.0403	1.01	0.98–1.04	0.4794
Emergency surgery	1.15	0.52–2.50	0.7192			

### LASSO analysis

**Figure 1:**
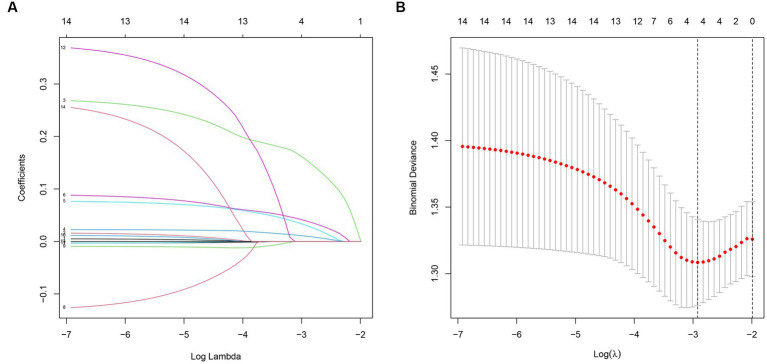
**(A)** The relationship between model AUC and log (λ) is shown by LASSO regression with 10-fold cross-validation. **(B)** LASSO regression (dashed line λ = 1 SE).

The LASSO regression with 10-fold cross-validation was performed on a training set of 150 patients. The results of the 10-fold cross-validation are shown in Figures 1A,B. The model achieved the maximum AUC when λ was set to the minimum mean squared error (λ min, 0.04905988) and included 4 variables. When λ was set to the minimum mean squared error plus one standard error (λ1 SE, 0.1133347), the model included no variables, but still achieved a high AUC. The relationship between the regression coefficients of each factor and λ is shown in Figure 1B. As λ increased, the regression coefficients gradually decreased. The final predictive model included NRS2002, ISS, APACHE II, and SOFA, with λ set at min (0.04905988).

### Nomogram

**Figure 2:**
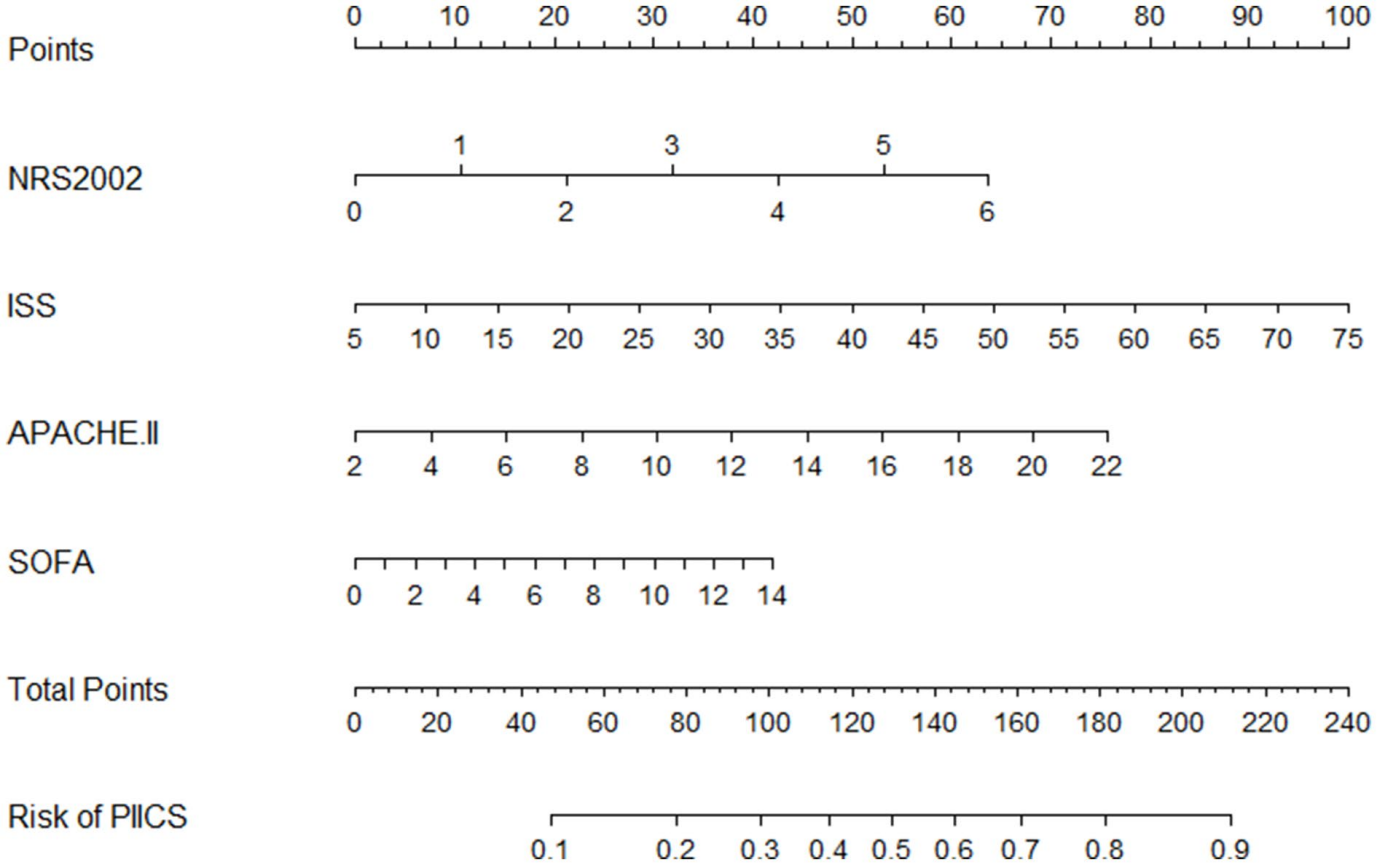
Nomogram prognostic model of PICS in patients with severe trauma during hospitalization.

A predictive model for the occurrence of PIICS during hospitalization in critically ill adult trauma patients was constructed using the selected factors (NRS2002, ISS, APACHE II, and SOFA). The model was presented in a nomogram (Figure 2). The nomogram showed that higher total scores were associated with a higher risk of PIICS. For example, if a critically ill trauma patient had NRS2002, ISS, APACHE II, and SOFA scores of 2, 20, 10, and 6 at admission, respectively, the corresponding scores on the nomogram were 21, 31, and 17, resulting in a total score of 90 and an estimated probability of developing PIICS during hospitalization of 27%.

### Predictive model performance

**Figure 3:**
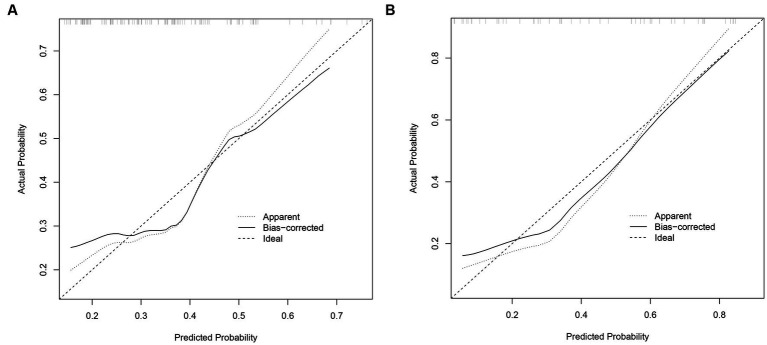
**(A)** Calibration diagram of the training set. **(B)** Calibration diagram of the validation set.

The performance of the prognostic model was evaluated through 1,000 bootstrapped samples to assess model calibration and potential overfitting. Calibration plots for PIICS prediction in the training and validation sets are shown in Figures 3A,B, respectively. The calibration of the PIICS model was assessed using the Hosmer-Lemeshow test. The results showed χ^2^ = 6.40, *p* = 0.699 for the training set and χ^2^ = 14.74, *p* = 0.098 for the validation set. Both value of ps were greater than 0.05, indicating an acceptable level of model fit.

### Differentiation

**Figure 4:**
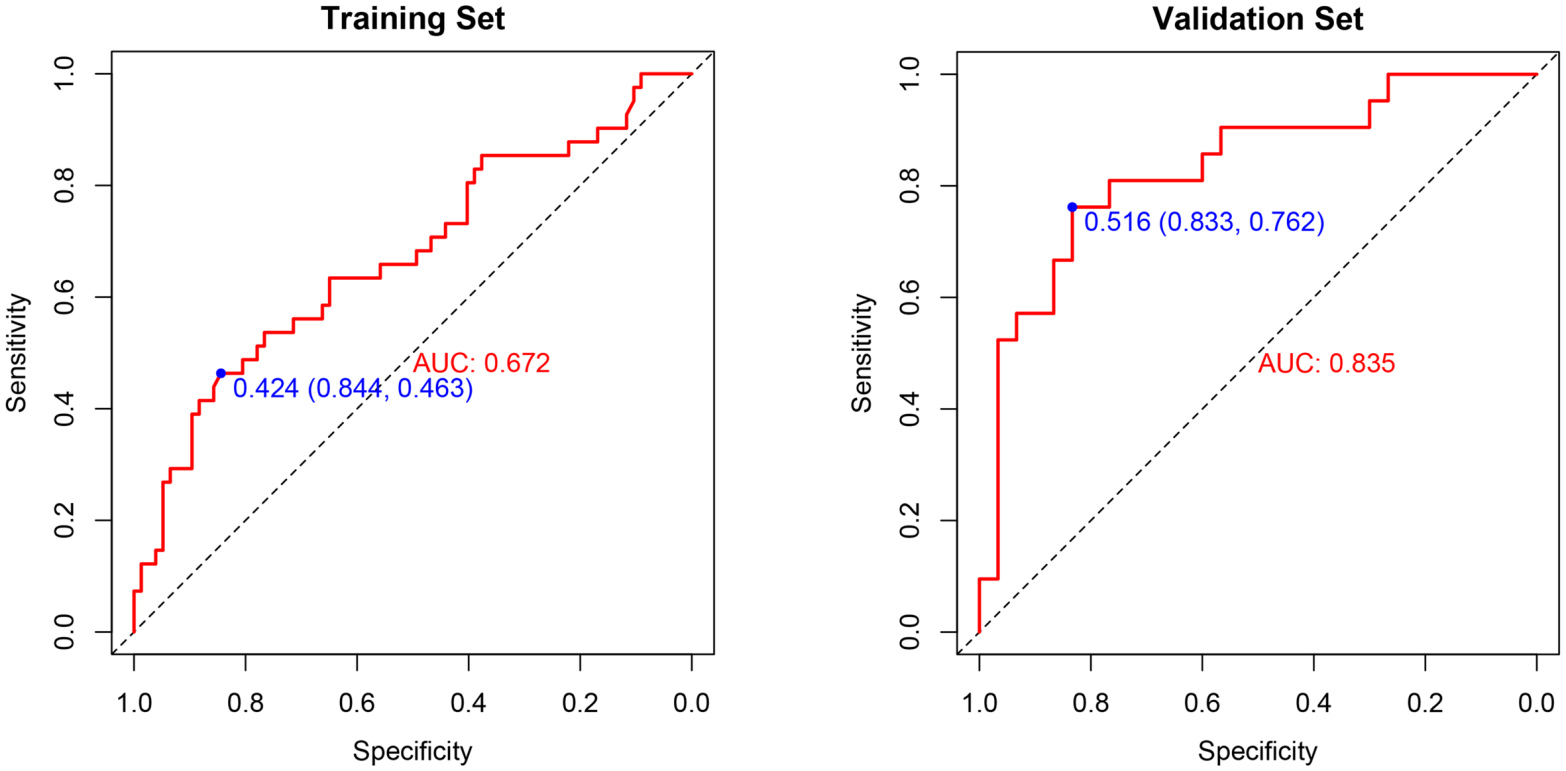
**(A)** ROC curve analysis of the prognostic model and various trauma scores in the training set. **(B)** ROC curve analysis of the prognostic model and various trauma scores in the validation set.

The ROC curves for the PIICS model in the modeling and validation sets of trauma patients are shown in Figures 4A,B, respectively. The discriminatory ability of the model was evaluated using the C-index. The C-index was 0.67 (95% CI 0.57–0.78) for the modeling set and 0.84 (95% CI 0.72–0.95) for the validation set, indicating a moderate discriminatory ability of the predictive model in both the training and validation sets.

### Decision curve analysis (DCA) curve

**Figure 5:**
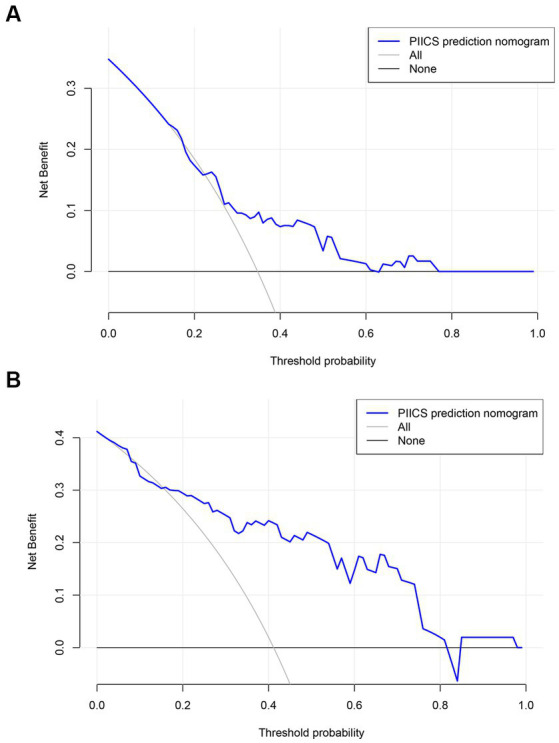
**(A,B)** The clinical decision curves showed that the prognostic model.

The clinical decision curves showed that the prognostic model had a considerable net benefit compared to the two extreme reference lines, both in the modeling and validation sets (Figures 5A,B). In the modeling set, the model had a higher net benefit when the risk threshold ranged from 0.15 to 0.62, while in the validation set, the model had a higher net benefit when the risk threshold ranged from 0.02 to 0.81.

## Discussion

In our study, we employed rigorous methods to develop and validate the nomogram, and obtaining a series of findings. (a) We retrospectively collected a large amount of data from severely traumatized patients and identified potential risk factors associated with the development of PIICS. These factors included demographic characteristics, injury severity scores, and other relevant clinical parameters. (b) Through multivariate analysis, we identified the most significant predictors and incorporated them into the nomogram. (c)The LASSO regression combined with 10-fold cross-validation was used to select four risk factors, including NRS2002, ISS, APACHE II, and SOFA, to construct a logistic model for predicting the risk of PIICS in severely traumatized patients. These findings provide an effective way to screen out PIICS early in our clinical work and may have a positive impact on patient care.

Various scoring systems with potential for predicting poor outcomes in severe trauma have been explored. NRS2002 is a tool for assessing nutritional risk in patients, considering factors such as nutritional intake, weight changes, and illness status to evaluate the level of nutritional risk (11). It has been reported to have predictive value in complications of severe trauma and is highly correlated with increased length of hospital stay (LOS) (12). ISS is a scoring system that assesses the severity of trauma based on the location and severity of injuries and is the most commonly used prognostic score in clinically severe multiple trauma patients (13, 14). APACHE II considers physiological indicators (e.g., blood pressure, respiratory rate, body temperature) and illness status (e.g., chronic diseases, age) to determine patient severity and prognosis (15) and has good performance in assessing in-hospital mortality in emergency trauma patients (16). The SOFA scoring system is used to assess the severity of multiorgan dysfunction in critically ill patients and has better performance in predicting mortality in both non-trauma and trauma patients (17, 18). However, individual scoring systems have limitations in predicting the complex complication of PIICS. Developing an early predictive model for PIICS is an important step in the prevention and management of complications in severe trauma (19). Therefore, an increasing number of studies have focused on exploring predictive factors for the occurrence of PIICS.

Different models have been developed for predicting fatigue syndrome and poor outcomes in elderly trauma patients (22), which have demonstrated good predictive and evaluative capabilities in trauma patients. By combining multiple variables and their respective weights, the nomogram provides a visualized model for risk prediction (23, 24), enabling clinicians to make effective predictions of the probability of PIICS occurrence in individual patients. This can aid in identifying high-risk patients and implementing targeted interventions, such as immunomodulatory therapy or nutritional support (25), to mitigate the progression of PIICS and its associated complications.

Compared to traditional prediction models or scoring systems, this model offers several advantages. Firstly, it incorporates a wide range of variables that capture the complexity of trauma patients developing PIICS. This comprehensive approach enhances the accuracy of risk prediction. Secondly, it provides a practical tool for clinicians to conduct real-time risk assessments in clinical practice. By inputting a patient’s clinical data into the model, an immediate estimation of the likelihood of PIICS development can be obtained, enabling early intervention.

Although our study has strengths, there are also limitations to consider. Firstly, the retrospective design introduces inherent biases and potential confounding factors. Prospective validation in well-designed cohorts would be valuable to confirm the generalizability of the nomogram. Secondly, our nomogram was developed and validated in a specific population of severely traumatized individuals, and its performance needs to be evaluated in other patient populations or healthcare settings. Furthermore, further validation in different centers or countries is required to ensure its applicability.

## Conclusion

In conclusion, the prognostic model developed in this study demonstrates good accuracy and discriminative ability. Developing and validating a predictive model specifically for PIICS in trauma patients is a crucial step toward personalized and proactive management of this complex syndrome. By utilizing existing data and analytical techniques, improving the prediction of risk stratification for severe trauma patients during hospitalization provides valuable insights for clinicians.

## Data availability statement

The raw data supporting the conclusions of this article will be made available by the authors, without undue reservation.

## Ethics statement

The studies involving humans were approved by this research plan has been approved by the Medical Ethics Committee of Tongji Hospital, Tongji Medical College, Huazhong University of Science and Technology (approval number: TJ-IRB20230214). The studies were conducted in accordance with the local legislation and institutional requirements. The participants provided their written informed consent to participate in this study.

## Author contributions

LX, ZK, DW, YL, and CW undertook the research, LX and YW wrote the main manuscript text and prepared figures. ZL, XB, and YW revised the article critically for important intellectual content and final approval of the version to be submitted. All authors contributed to the design of the study and the writing of the manuscript and reviewed the manuscript.

## Funding

This study was supported by grants from the National Natural Science Foundation of China (Grant No. 82002101).

## Conflict of interest

The authors declare that the research was conducted in the absence of any commercial or financial relationships that could be construed as a potential conflict of interest.

## Publisher’s note

All claims expressed in this article are solely those of the authors and do not necessarily represent those of their affiliated organizations, or those of the publisher, the editors and the reviewers. Any product that may be evaluated in this article, or claim that may be made by its manufacturer, is not guaranteed or endorsed by the publisher.
